# Prevalence of Thyroid Transcription Factor-1 (TTF-1)-Negative Small Cell Carcinoma and Napsin A Positivity in Small Cell Carcinoma in a Cross-Sectional Study of Lung Core Biopsies

**DOI:** 10.7759/cureus.37015

**Published:** 2023-04-01

**Authors:** Shahistha Sayeda, Asghar Naqvi, Housne Begum, Rosalyn A Juergens, Christian Finley, Courtney H Coschi, Jean-Claude Cutz, Michael Bonert

**Affiliations:** 1 Pathology and Molecular Medicine, McMaster University, Hamilton, CAN; 2 Thoracic Surgery, McMaster University, Hamilton, CAN; 3 Oncology, McMaster University, Hamilton, CAN; 4 Surgery, Division of Thoracic Surgery, McMaster University, Hamilton, CAN; 5 Medical Oncology, McMaster University, Hamilton, CAN

**Keywords:** small cell lung carcinoma, ki- 67, napsin a, ttf-1, lung core biopsies

## Abstract

Background

The prevalence of thyroid transcription factor-1 (TTF-1) and napsin A expression are poorly characterized in lung core biopsies of small cell carcinoma. Locally, the TTF-1 clone is 8G7G3/1 (Agilent/Dako), and the napsin A clone is IP64 (Leica Biosystems).

Methods

All in-house lung core biopsy reports for cases accessioned at a regional laboratory from January 2011 to December 2020 were retrieved and analyzed using a validated hierarchical free-text string matching algorithm (HFTSMA) to establish the diagnosis. TTF-1 and napsin A were manually coded with the assistance of a logical text parsing tool. All TTF-1-negative small cell lung carcinoma (SCLC) cases had a full report review by pathologists.

Results

The cohort had 5,867 lung core biopsies, and 232 cases were confirmed as small cell carcinoma on pathologist review. TTF-1 immunostain results were available in 173 SCLC cases, and 16 cases of TTF-1-negative SCLC were confirmed on full report review. These 16 cases had at least one positive neuroendocrine (NE) marker and positive keratin staining; cases with mixed histology or positive CK5/6 staining were excluded. Ki-67 was done in 10/16 cases; the average Ki-67 was 75%. Napsin A was negative in 50/51 small cell carcinomas, and 0/3 TTF-1-negative SCLC had napsin A positivity.

Conclusions

Standardized immunostain reporting would simplify such analyses. Based on the cohort, approximately 9% (16/173) of SCLC is TTF-1 negative. Napsin A positivity in suspected small cell carcinoma should prompt consideration of an alternate diagnosis or explanation.

## Introduction

Small cell lung carcinoma (SCLC) is relatively less common; it represents approximately 15% of lung cancers [1,2]. It has a strong association with cigarette smoking, and most cases are diagnosed at an advanced stage. The diagnosis is usually a constellation of histo/cytomorphology along with immunohistochemical workup. The typical immunohistochemical workup includes neuroendocrine (NE) markers, thyroid transcription factor-1 (TTF-1), and proliferation marker (Ki-67). SCLC is typically TTF-1 positive; however, it has only been studied in modest-size cohorts [3,4]. The classic study by Folpe et al., using the clone 8G7G3/1 (Agilent/Dako), showed that TTF-1 was positive in 20 of 21 SCLCs [5]. A study by Misch et al. using the SP141 TTF-1 clone (Ventana Systems) found 38 of 221 SCLC patients had TTF-1-negative tumors and that the TTF-1 status did not predict survival [3]. A case-control study by Iida et al., with 11 TTF-1-negative SCLCs and 24 TTF-1-positive SCLCs using the SPT24 TTF-1 clone (Leica Biosystems), suggested that TTF-1-negative SCLCs express NE markers less frequently and, like Misch et al., do not have a significantly different survival between groups [4].

It should be noted that TTF-1 staining is clone dependent [6,7] and not entirely specific for the lung (or thyroid); TTF-1 may be positive in non-SCLCs [8] and maybe positive in non-lung cancers, e.g., colorectal cancer [9], endometrial cancer [10], and lymphoma [6].

Napsin A is a commonly used immunostain in the workup of suspected primary lung adenocarcinoma. It is typically positive in lung adenocarcinoma [11]; however, the literature on napsin A expression in lung NE neoplasms is limited. A series of 37 SCLC surgical resection cases were all negative for napsin A [12]; likewise, a series of 36 cytology cases of SCLC were all napsin A negative. [13]. A larger review of napsin A staining suggested positivity of 0%-17% in (lung) small cell carcinoma [11]. Napsin A expression has been examined in a series of 112 large-cell NE carcinomas; in that context, napsin A is positive in 15% of cases [12]. In medium-size pathology practices without sub-specialization and a modest volume of lung biopsies, small cell carcinoma may be a diagnosis that is seen by the individual pathologist once or twice a year. In such environments, where the working diagnosis is small cell carcinoma but the TTF-1 immunostain is negative, an external consultation may be sought due to uncertainty about the tumor sub-type. Prior (unpublished) work showed that individual pathologists in our thoracic referral center diagnose only two to three small cell carcinomas on lung core biopsies per year.

Objective

The primary objective of this study was to estimate the number of TTF-1-negative SCLCs in a large lung core biopsy cohort. The secondary objective was to assess the prevalence of napsin A staining in all small cell carcinomas.

## Materials and methods

Research ethics board approval was obtained to retrieve lung pathology reports; Hamilton Integrated Research Ethics Board (HiREB) issued approval 3811. The TTF-1 clone in use is 8G7G3/1 (Agilent/Dako), and the napsin A clone is IP64 (Leica Biosystems). All in-house lung core biopsy reports for cases accessioned at a regional laboratory from January 2011 to December 2020 were retrieved using complex search criteria (to exclude open lung biopsies and resections) and analyzed using a validated hierarchical free-text string matching algorithm (HFTSMA) to establish the diagnosis. The diagnostic codes used to classify cases and the hierarchy are defined in Appendix A.

**Figure 1:**
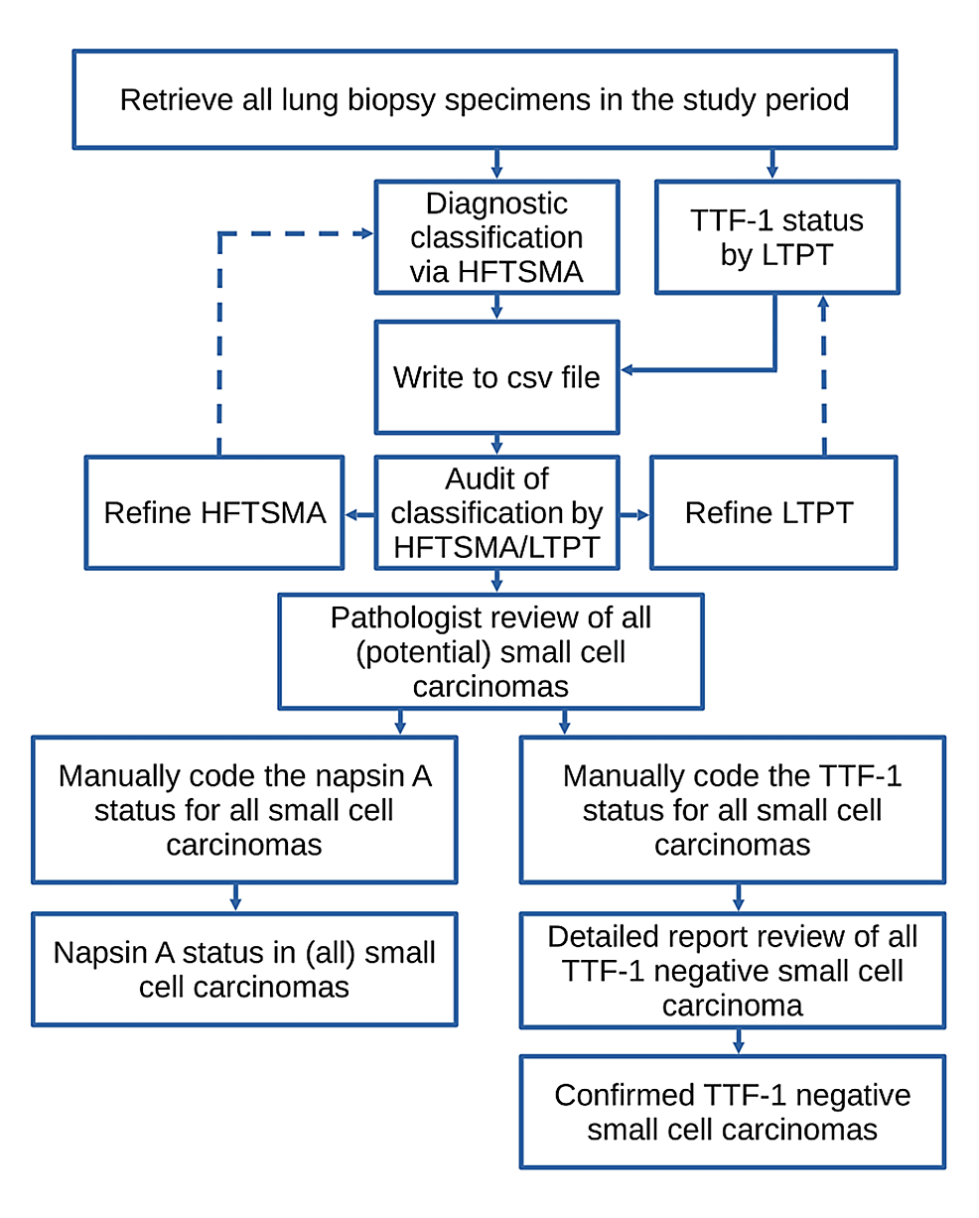
Overview of methods HFTSMA: Hierarchical free-text string matching algorithm; TTF-1: Thyroid transcription factor-1; LTPT: Logical text parsing tool.

This work builds on an abstract presented at the United States and Canadian Academy of Pathology Annual Meeting in Los Angeles, USA, 2020, and an abstract presented at the European Congress of Pathology in Basel, Switzerland, 2022. An overview of the methods is shown in Figure 1.

A list of immunostains (see Appendix B) was coded by a logical text parsing tool (LTPT) for all lung biopsies. The LTPT separated sentences and determined the immunostain interpretation based on its location in the sentence in relation to phrases for "negative" and "positive." The LTPT classifications were reviewed (and corrected if necessary) by a pathologist reviewing the report text of all (possible) small cell carcinomas identified by the HFTSMA. All possible TTF-1-negative small cell carcinoma cases had a full report review by two pathologists (AN and MB).

The inclusion criteria for cases in this study were: (1) cases reported as "small cell carcinoma," (2) TTF-1-negative cases with at least one reported positive NE marker, and (3) reported positive keratin staining (CAM5.2 or AE1/AE3 or CK7). Cases with mixed histology or positive CK5/6 staining were excluded.

## Results

The cohort had 5,867 lung core biopsies that came from 4,973 patients. The HFTSMA categorized 5,725 cases (98%). A total of 254 cases were identified as possible small cell carcinoma by the HFTSMA, and 232 cases were classified as small cell carcinoma on a pathologist review of the reports. Cases were excluded if the diagnosis in the report was not unambiguously small cell carcinoma or suggested a mixture of histologic types (15 cases). TTF-1 immunostain results were available in 173 of the 232 cases of confirmed small cell carcinoma. TTF-1 negative cases were excluded if no keratin positivity was reported (seven cases).

TTF-1-negative small cell carcinomas

Sixteen cases of SCLC were confirmed in the full report review. These 16 had at least one positive NE marker (chromogranin A, synaptophysin, CD56) and positive keratin staining with CAM5.2, CK7, or AE1/AE3; cases with mixed histology or positive CK5/6 staining were excluded. CAM5.2 was positive in 13 of 13 cases. CK7 was positive in seven of nine cases. Keratin staining was described in the report as dots in three cases. All cases (16/16) were CD56 positive. Six cases had two NE markers positive, and three cases had all three NE markers positive. Seven were stained for CK20 and were negative. The results of the case are shown in Table 1.

**Table 1:** Immunostaining results for the 16 confirmed TTF-1-negative small cell carcinomas NA: Not available; TTF-1: Thyroid transcription factor-1.

Case	TTF-1	Napsin A	CK5/6	p63	p40	CD56	Chromogranin A	Synaptophysin	AE1/AE3	CK7	CK20	GATA3	CAM5.2	PSA	CD45
Case 1	-	NA	NA	-	NA	+	-	+	NA	-	-	NA	+	NA	-
Case 2	-	NA	NA	NA	NA	+	+	NA	NA	NA	NA	NA	+	NA	NA
Case 3	-	NA	NA	-	NA	+	NA	+	NA	+	-	NA	+	NA	-
Case 4	-	NA	NA	-	NA	+	+	-	NA	+	NA	NA	+	NA	NA
Case 5	-	NA	NA	-	NA	+	+	+	NA	NA	NA	NA	+	NA	NA
Case 6	-	NA	NA	NA	NA	+	NA	NA	NA	+	-	NA	NA	NA	NA
Case 7	-	NA	NA	NA	NA	+	NA	NA	NA	NA	NA	NA	+	NA	NA
Case 8	-	NA	NA	NA	NA	+	NA	NA	+	NA	NA	NA	NA	NA	NA
Case 9	-	NA	NA	-	NA	+	NA	+	NA	+	-	NA	+	NA	-
Case 10	-	NA	NA	-	NA	+	NA	NA	NA	NA	NA	NA	+	NA	NA
Case 11	-	NA	NA	+	NA	+	NA	NA	NA	NA	NA	NA	+	NA	NA
Case 12	-	-	-	-	NA	+	+	+	NA	+	-	NA	NA	NA	NA
Case 13	-	NA	-	-	NA	+	-	-	+	-	NA	NA	+	NA	-
Case 14	-	-	-	+	NA	+	NA	+	+	NA	NA	NA	+	NA	NA
Case 15	-	NA	-	+	NA	+	-	-	NA	+	-	-	+	-	NA
Case 16	-	-	-	NA	-	+	+	+	NA	+	-	NA	+	NA	NA
Total negative	16	3	5	8	1	0	3	3	0	2	7	1	0	1	4
Total positive	0	0	0	3	0	16	5	7	3	7	0	0	13	0	0
Total NA	0	13	11	5	15	0	8	6	13	7	9	15	3	15	12
Sum	16	16	16	16	16	16	16	16	16	16	16	16	16	16	16
% positive	0%	0%	0%	27%	0%	100%	63%	70%	100%	78%	0%	0%	100%	0%	0%

Ki-67 was done in 10/16 cases; the average Ki-67 was 75%. The average number of immunostains per case was 7.3 immunostains (range: 3-13). Results by the case are shown in Table 2.

**Table 2:** Selected results for the 16 confirmed TTF-1-negative small cell carcinoma CK: Cytokeratin; TTF-1: Thyroid transcription factor-1; NA: Not available; HR: Heat retrieval; this roughly corresponds to the total number of immunostains done on the case.

Case	HR	Ki-67 (%)	Dot-like CK
Case 1	8	55	Yes
Case 2	5	90	NA
Case 3	7	75	Yes
Case 4	8	60	NA
Case 5	6	NA	NA
Case 6	5	80	NA
Case 7	3	NA	NA
Case 8	5	90	NA
Case 9	9	NA	NA
Case 10	5	NA	NA
Case 11	6	60	NA
Case 12	11	80	NA
Case 13	13	NA	Yes
Case 14	9	75	NA
Case 15	13	80	NA
Case 16	4	NA	NA
Average	7.3125	74.5	NA
Median	6.5	77.5	NA
Max	13	90	NA
Min	3	55	NA

Napsin A staining

Napsin A staining status by the LTPT with pathologist review demonstrated that only 1/51 case was napsin A positive. The napsin A positive case was TTF-1/CD56/synaptophysin (diffuse and strong) positive; chromogranin A was focal and weak; p63 and CK5/6 were negative; Ki67 proliferation index was 90%, and the napsin staining was described as "very rare cells showing cytoplasmic staining with napsin A." All TTF-1-negative small cell carcinomas with reported napsin A staining were napsin A negative (0/3) (Table 1).

## Discussion

The findings suggest that TTF-1-negative small cell carcinoma of the lung is an infrequent occurrence. In our environment, it is seen approximately two times a year on lung core biopsy. In environments where lung core biopsies are relatively less frequent, finding that a presumed small cell carcinoma is TTF-1 negative may be disconcerting and may prompt an external review.

In relation to Iida et al., the cohort in this study had more NE marker positivity; this difference may be explained by the different TTF-1 clones used (SPT24 versus 8G7G3/1) [4]. It should be noted that the International Association of Lung Cancer Study (IASLC) has criteria for TTF-1 positivity [14]. It is presumed that these were applied in the routine in-house practice; however, this was not assessed. Similarly, napsin A staining was presumed to be assessed based on positive internal controls (cytoplasmic expression in type II pneumocytes and alveolar macrophages).

Lung tumors with morphology suggestive of small cell carcinoma that is negative for TTF-1 should prompt consideration of a wider differential diagnosis. The differential diagnosis of SCLC includes other primary NE tumors (typical carcinoid, atypical carcinoid, and large cell NE carcinoma), basaloid squamous cell carcinoma, combined adenocarcinoma, small cell carcinoma, small round cell sarcomas both in the Ewing sarcoma family (e.g., Ewing sarcoma) and recently described morphologically similar tumors lacking EWSR1 gene rearrangement (e.g., CIC-DUX4-rearranged and BCOR-CCNB3-rearranged tumors), Merkel cell carcinoma, lymphomas, thoracic SMARCA4-deficient undifferentiated tumors (SMARCA4-UT) with small round cell morphology, NUT carcinoma, and metastasis from small cell carcinoma of HPV-related sites (cervix, head, and neck) [15-17]. Ki-67 immunostaining is often very helpful in differentiating NE tumor types, especially in small biopsies [18]. In the context of negative TTF-1 staining, keratin positivity, NE marker positivity, and clinical history are important to make an accurate diagnosis. It should be noted that many tumors have a small cell variant, so there should be a low threshold for consultation with an expert [1].

Napsin A negativity in small cell carcinoma was seen in 98% of cases (50/51). Napsin A was negative in all three TTF-1-negative small cell carcinomas, where a napsin A result was available. The one case that was napsin A positive was reviewed by a fellowship-trained lung pathologist and had an immunoprofile compatible with small cell carcinoma except for the reported napsin A staining. Napsin A positivity, in the context of suspected small cell carcinoma, appears to be very uncommon. We believe napsin A positivity should prompt strong consideration of alternative diagnoses or an alternative explanation, such as napsin A staining in non-tumor cells that are in the background. One consideration is mixed non-small cell and small cell carcinoma. Given the advances in biomarker testing in non-small cell carcinoma (e.g., epidermal growth factor receptor [EGFR], anaplastic lymphoma kinase [ALK], programmed death ligand-1 [PD-L1], ROS-1), testing should be done if a non-small carcinoma component cannot be excluded​​​​.** **Small cell carcinomas can evolve de novo or from non-small cell carcinoma; the clinical history and prior pathology may provide important clues that a case has evolved from non-small cell carcinoma and should get tested for drug-able molecular mutations/non-SCLC biomarkers.

## Conclusions

Useful information can be extracted from free-text pathology reports using text processing programs; however, auditing is required to ensure accurate classification. Standardized immunostain reporting would simplify such analyses. Based on the available data in the cohort, napsin A positivity with clone IP64 in small cell carcinoma is very rare and should prompt consideration of alternate diagnoses. Approximately 9% (16/173) of SCLC is TTF-1 negative with the clone 8G7G3/1.

## Appendices

Appendix A 

**Table 3:** Diagnostic codes and text

Code	Text
dx2	Adenocarcinoma
dx3	Squamous carcinoma
dx3	Squamous cell carcinoma
dx4	Small cell carcinoma
dx4	Small cell neuroendocrine carcinoma
dx5	Non-small cell carcinoma
dx5	Non small cell carcinoma
dx5	Nonsmall cell carcinoma
dx6	Large cell neuroendocrine carcinoma
dx7	Atypical carcinoid
dx8	Typical carcinoid
dx8	Carcinoid
dx9	Atypical mesothelial hyperplasia
dx10	Mesothelioma
dx15	Atypical glandular proliferation
dx15	Atypical adenomatous hyperplasia
dx15	Atypical alveolar hyperplasia
dx15	Atypical alveolar cells
dx15	Atypical bronchioalveolar proliferation
dx20	Malignant cells
dx20	Malignant tumor
dx20	Malignant tumour
dx20	Malignant neoplasm
dx20	Small blue cell tumor
dx20	Small blue cell tumour
dx20	Small round blue cell tumour
dx20	Poorly differentiated neoplasm
dx20	Malignant epithelial neoplasm
dx20	Malignant glands seen
dx20	Malignant glandular neoplasm
dx20	Invasive epitheloid neoplasm
dx20	Positive for epithelial neoplasm
dx20	Neuroendocrine neoplasm
dx20	Myxoid neoplasm
dx20	Clear cell neoplasm
dx21	Lymphoproliferative disorder
dx21	Lymphoproliferative process
dx21	Monotonous lymphoid infiltrate
dx21	Atypical lymphoid aggregate
dx21	Atypical lymphoid infiltrate
dx21	Suggestive of lymphocytic proliferation
dx21	Suspicious for lymphoplasmacytic proliferative disorder
dx21	Dense lymphocytic infiltrative proliferation
dx22	Lymphoma
dx23	Sarcoma
dx23	Sarcoma,
dx25	Soft tissue neoplasm
dx25	Spindle cell lesion
dx25	Spindle cell neoplasm
dx27	Suspect for neoplasm
dx27	Atypical cells identified
dx27	Atypical cells present
dx27	Few atypical cells
dx27	Atypical epithelioid cells
dx27	Atypical alveolar epithelial cells
dx27	Atypical glands
dx27	Atypical cells
dx27	Focal atypical change
dx27	Atypical infiltrate
dx27	Atypical glandular epithelium
dx27	Atypical squamous epithelial
dx27	Atypical squamous epithelium
dx27	Atypical bronchioalveolar lining cells
dx27	Atypical TTF-1 positive cells
dx27	Minimal cytologic atypia
dx27	Suspicious for a neoplasm
dx27	Suspicious for malignancy
dx27	Suspect for malignancy
dx27	Suggestive of malignancy
dx27	Cannot rule out malignancy
dx28	Suspicious for a non-Hodgkin's lymphoma
dx28	Suspicious for non-small cell carcinoma
dx28	Suspicious for non small cell carcinoma
dx28	Suspicious for nonsmall cell carcinoma
dx28	Suspicious for poorly differentiated carcinoma
dx28	Suspicious for small cell carcinoma
dx28	Suspicious for adenocarcinoma
dx28	Suspicious for squamous cell carcinoma
dx28	Suspicious for squamous carcinoma
dx28	Suspicious for lymphoma
dx28	Suspicious for carcinoma
dx29	Pulmonary adenocarcinoma
dx29	Lung primary
dx30	Metastatic
dx30	Metastasis
dx31	Colon
dx31	Colonic
dx31	Rectal
dx31	Colorectal
dx32	Breast
dx33	Urothelial carcinoma
dx33	Urothelial cell carcinoma
dx33	Transitional cell carcinoma
dx33	Bladder
dx34	Prostatic origin
dx34	Prostate
dx35	Thyroid
dx36	Melanoma
dx37	Kidney
dx37	Renal
dx40	Thymoma
dx41	Sclerosing hemangioma
dx41	Pneumocytoma
dx50	Carcinoma
dx60	Necrosis
dx60	Necrotic
dx60	Necrotizing inflammation
dx61	Respiratory bronchiolitis interstitial lung disease
dx61	RBILD
dx61	Smoker's respiratory bronchiolitis
dx61	Features of respiratory bronchiolitis
dx65	Granulation tissue
dx66	Squamous papilloma
dx67	Vegetable matter
dx67	Foreign plant material
dx67	Plant origin
dx70	Benign-appearing bronchial tissue
dx70	Benign bronchial tissue
dx70	Unremarkable bronchial mucosa
dx70	Benign bronchial mucosa
dx70	Benign bronchial epithelium
dx70	Bronchial mucosa with no pathologic finding
dx70	Benign-appearing respiratory mucosa
dx70	Benign respiratory mucosa
dx70	Unremarkable endobronchial tissue
dx70	Unremarkable endobronchial mucosa
dx70	Unremarkable bronchial wall tissue
dx70	Unremarkable tiny fragments of endobronchial mucosa
dx70	Benign respiratory epithelium
dx70	Respiratory epithelium without significant diagnostic abnormality
dx70	Unremarkable fragments of cartilage and endobronchial mucosa
dx70	Unremarkable cartilage and bronchial tissue
dx70	Benign bronchial wall
dx70	Bronchial mucosa without findings
dx70	Benign chronic inflamed respiratory mucosa
dx70	Benign, chronic inflamed bronchial mucosa
dx70	Benign edematous bronchial tissue
dx70	Edematous bronchial mucosa with no other specific pathology
dx70	Benign pulmonary parenchyma
dx70	Unremarkable lung parenchyma
dx70	Benign lung tissue
dx70	Benign alveolar lung tissue
dx70	Benign alveolar lung parenchyma
dx70	Benign-appearing lung parenchyma
dx70	Benign lung parenchyma
dx70	Benign parenchymal lung tissue
dx70	Lung parenchymal tissue with no significant histological abnormality
dx70	Bronchial and lung alveolar tissue without specific diagnostic abnormality
dx70	Benign bronchial and alveolar tissue
dx70	Benign bronchial and alveolar lung tissue
dx70	Benign parenchymal lung
dx70	Benign lung tissue
dx70	Benign bronchial parenchyma
dx70	Benign respiratory epithelial cells
dx70	Benign lung and bronchial parenchyma
dx70	Alveolar tissue with no significant findings
dx70	Alveolar lung tissue without diagnostic abnormality
dx70	Benign soft tissue
dx70	Very small amount of bland epithelium
dx70	Benign fibroadipose tissue and blood
dx70	Alveolar tissue without significant findings
dx70	Mild chronic inflammation, no specific findings
dx70	Minimal chronic inflammation, no other findings
dx70	Chronic inflammation
dx70	Lung-benign
dx70	Benign crushed respiratory mucosa
dx70	Lung parenchyma with reactive pneumocyte proliferation
dx70	Thickened fibrous pleura
dx70	Consistent with fibrinous pleuritis
dx70	Unremarkable lymphoid tissue and lung parenchyma
dx71	Organized pneumonia
dx71	Organizing pneumonia
dx71	Bronchopneumonia with focal organization
dx72	Granuloma
dx72	Granulomatous inflammation
dx73	Nonspecific scar
dx73	Suggestive of scar
dx73	Scarred lung tissue
dx73	Scarred alveolar lung
dx73	Scarlike tissue
dx73	Scarring
dx73	Scarred lung
dx73	Scar tissue
dx73	Dense scar
dx73	Fibroelastotic scar
dx73	Bland fibrosis
dx74	Hamartoma
dx75	Solitary (isolate) fibrous tumour
dx75	Solitary fibrous tumour
dx75	Solitary fibrous tumor
dx75	Solitary/localized fibrous tumor
dx76	Pleural plaque
dx77	Langerhans cell histiocytosis
dx78	Amyloid
dx79	Lymphocytic infiltrate, favor reactive or inflammatory process
dx79	Lymphocytic infiltrate, favour benign
dx79	Benign, reactive lymphoid proliferation
dx79	Benign lymph nodal tissue
dx79	Bronchial mucosa showing mild acute and chronic inflammation
dx79	Bronchial mucosa with mild chronic inflammation and some cartilage
dx79	Bronchial mucosa with mild chronic inflammation
dx79	Endobronchial mucosa with mild chronic inflammation
dx79	Lung alveolar tissue showing mild chronic inflammation
dx79	Bronchial tissue showing chronic inflammation
dx79	Bronchial tissue showing mild chronic inflammation
dx79	Respiratory mucosa with mild chronic inflammation
dx79	Lung parenchyma with fragment of cartilage and calcification
dx79	Benign-appearing fibroadipose tissue
dx79	Bronchial mucosa with eosinophilia
dx79	Lung tissue with bronchial dilatation associated with acute and inflammation
dx79	Nonspecific chronic interstitial pneumonia
dx79	Ulceration and acute inflammation
dx79	Chronic inflamed bronchial mucosa
dx79	Bronchial mucosa with squamous metaplasia and acute inflammation
dx79	Chronic bronchiolitis
dx80	No diagnostic abnormality
dx80	No significant pathology
dx80	No specific pathology
dx80	No definite pathological diagnosis
dx80	No histopathological abnormality
dx80	No significant abnormality
dx80	No significant findings
dx80	No findings
dx80	No significant pathological abnormality
dx80	No significant pathological changes
dx80	No significant histological abnormality
dx80	No specific pathologic finding
dx80	No pathologic diagnosis
dx80	No apparent lesional tissue
dx80	No lesional tissue
dx80	Without significant pathology
dx80	Negative for significant pathology
dx80	Without significant histopathologic abnormality
dx81	No granuloma
dx81	Negative for granuloma
dx82	Negative for thymoma
dx82	Negative for carcinoma or thymoma
dx90	Negative for malignancy
dx90	Negative for evidence of malignancy
dx90	No tumor or malignancy
dx90	No malignancy identified
dx90	No malignancy in these biopsies
dx90	No malignancy is seen
dx90	No evidence of malignancy
dx90	No evidence of dysplasia and malignancy
dx90	No definite evidence of malignancy
dx90	No malignancy is identified
dx90	No evidence of dysplasia or malignancy
dx90	Negative for dysplasia or malignancy
dx90	Negative for dysplasia and malignancy
dx90	No evidence of inflammation, dysplasia or malignancy
dx90	Negative for specific inflammation, benign or malignant neoplasm
dx90	Negative for epithelial malignancy
dx90	Negative for carcinoma
dx90	No evidence of carcinoma
dx91	No obvious epithelial neoplasm identified
dx91	Negative for lung tissue or neoplasm
dx91	Negative for neoplasia
dx91	No evidence of neoplasia
dx91	No evidence of a tumour
dx91	No tumor present
dx91	No tumor identified
dx91	No tumors identified
dx91	Negative for neoplasm
dx91	No evidence of cyst or neoplasm
dx91	No neoplastic tissue
dx91	No evidence of viable neoplasia
dx92	No evidence of metastatic malignancy
dx92	No evidence of metastatic disease
dx92	Negative for metastatic malignancy
dx92	No metastatic disease
dx92	No evidence of metastatic malignancy
dx92	No evidence of metastasis
dx92	Negative for metastasis
dx93	No evidence of metastatic or granulomatous disease
dx93	No evidence of granulomatous disease or metastatic malignancy
dx93	Negative for granulomatous disease or metastatic malignancy
dx93	Negative for granuloma or malignancy
dx93	Negative for malignancy, granulomas
dx93	Negative for metastatic malignancy or granulomatous disease
dx95	Likely not representative
dx95	May not be representative
dx95	May not be a representative tissue
dx95	Not representative of the lesion
dx95	Clinical correlation required regarding representation
dx95	? Representative
dx95	No kidney parenchyma
dx95	No kidney tissue present
dx99	Not specific
dx99	No specific diagnosis
dx99	Non-diagnostic
dx99	Non diagnostic
dx99	Not diagnostic
dx99	Unsatisfactory specimen
dx99	Not further diagnostic
dx99	Markedly degenerate sample
dx99	Material not assessable
dx99	Insufficient tissue for pathologic evaluation
dx99	No diagnostic material
dx99	No diagnostic lung tissue
dx99	Nondiagnostic tissue
dx99	No viable lung tissue present
dx99	No viable tissue identified
dx99	Crushed urothelial tissue
dx99	Scanty crushed tissue
dx99	Insufficient for diagnosis
dx99	Insufficient tissue for diagnosis
dx99	Material insufficient
dx99	Insufficient tissue for assessment
dx99	Insufficient for assessment
dx99	Insufficient material
dx99	Inconclusive
dx99	Tissue insufficient
dx99	Tissue did not survive the processing
dx99	No tissue material
dx99	No tissue is seen at microscopy
dx99	Material insufficient for assessment
dx99	No lung tissue present for evaluation
dx99	Lung-skeletal muscle and fibrofatty tissue

Appendix B 

**Table 4:** List of immunostains parsed by the logical text parsing tool

Immunostain	Search strings
TTF-1	"TTF-1", "TTF1"
Napsin A	"napsin", "Napsin"
CK5/6	"cytokeratin 5/6", "CK 5/6", "CK5/6"
p63	"p63", "P63"
p40	"p40", "P40"
CD56	"CD56"
Chromogranin A	"chromogranin"
Synaptophysin	"synaptophysin"
AE1/AE3	"AE1/AE3", "AE1 AE3", "AE1/3"
CK7	"cytokeratin 7", "CK 7", "CK7", "Ck7"
CK20	"cytokeratin 20", "CK 20", "CK20", "Ck20"
GATA3	"GATTA3", "GATA3"
CDX2	"CDX 2", "CDX2"
PAX8	"PAX 8", "PAX-8", "PAX8"
S100	"S100", "S-100"
HepPar-1	"HepPar", "Hep Par", "HEPPAR", "HEP PAR"
CA-125	"CA125", "CA-125"
CAM 5.2	"CAM 5", "CAM5", "cam5", "Cam5", "Cam 5"
Mammaglobin	"mammaglobin"
Thyroglobulin	"thyroglobulin"
PSA	"PSA"
Calretinin	"calretinin"
Melan A	"melan ", "MELAN "
HMB-45	"HMB"
Vimentin	"vimentin", "VIMENTIN"
ER	" ER,", " ER "
PR	"PR"
HER2	"HER2", "HER-2", "Her2", "Her-2"
BRST2	"BRST2", "BRST-2", "GCDFP"

Appendix C 

**Table 5:** Hierarchy of diagnostic codes NSCLC: Non-small cell lung carcinoma; SCC: Small cell carcinoma; NSCC: Non-small cell carcinoma; NOS: Not otherwise specified.

Supersedes	Precedes	Meaning
dx5	dx4	Non-small cell ca over small cell carcinoma
dx2	dx5	Adenoca over NSCLC
dx3	dx5	SCC over NSCLC
dx2	dx50	Adenoca over carcinoma
dx3	dx50	SCC over carcinoma
dx4	dx50	SmCC over carcinoma
dx5	dx50	NSmCC over carcinoma
dx7	dx8	Atypical carcinoid over typical carcinoid
dx20	dx28	Malignant over suspicious for X
dx90	dx20	Neg for malignant cells over malignant cells
dx2	dx20	Adenoca over malignant
dx3	dx20	SCC over malignant
dx4	dx20	SmCC over malignant
dx5	dx20	NSmCC over malignant
dx70	dx27	Benign/favor reactive over atypical
dx92	dx30	Neg for met over met
dx93	dx30	Neg for met+granuloma over met
dx93	dx72	Neg for met+granuloma over granuloma
dx90	dx50	Neg for malignancy/carcinoma over carcinoma NOS
dx28	dx22	Susp for lymphoma over lymphoma
dx28	dx2	Susp for adenoca over adenoca
dx28	dx3	Susp for SCC over SCC
dx28	dx5	Susp for NSCC over NSCC
dx28	dx4	Susp for SmCC over SmCC
dx28	dx50	Susp for carcinoma over carcinoma NOS
dx28	dx27	Susp for X over atypical cells/less specific atypical
dx81	dx72	No granuloma over granuloma
dx82	dx40	Neg for thymoma over thymoma
